# Single Cell Transcriptome Analysis of Peripheral Blood Mononuclear Cells in Freshly Isolated versus Stored Blood Samples

**DOI:** 10.3390/genes14010142

**Published:** 2023-01-04

**Authors:** Hui-Qi Qu, Charlly Kao, James Garifallou, Fengxiang Wang, James Snyder, Diana J. Slater, Cuiping Hou, Michael March, John J. Connolly, Joseph T. Glessner, Hakon Hakonarson

**Affiliations:** 1 The Center for Applied Genomics, Children’s Hospital of Philadelphia, Philadelphia, PA 19104, USA; 2 Department of Pediatrics, The Perelman School of Medicine, University of Pennsylvania, Philadelphia, PA 19104, USA; 3 Division of Human Genetics, Children’s Hospital of Philadelphia, Philadelphia, PA 19104, USA; 4 Division of Pulmonary Medicine, Children’s Hospital of Philadelphia, Philadelphia, PA 19104, USA; 5 Faculty of Medicine, University of Iceland, 101 Reykjavik, Iceland

**Keywords:** AP-1, NF-kB, peripheral blood mononuclear cells, single cell RNA-seq

## Abstract

Background: Peripheral blood mononuclear cells (PBMCs) are widely used as a model in the study of different human diseases. There is often a time delay from blood collection to PBMC isolation during the sampling process, which can result in an experimental bias, particularly when performing single cell RNA-seq (scRNAseq) studies. Methods: This study examined the impact of different time periods from blood draw to PBMC isolation on the subsequent transcriptome profiling of different cell types in PBMCs by scRNAseq using the 10X Chromium Single Cell Gene Expression assay. Results: Examining the five major cell types constituting the PBMC cell population, i.e., CD4+ T cells, CD8+ T cells, NK cells, monocytes, and B cells, both common changes and cell-type-specific changes were observed in the single cell transcriptome profiling over time. In particular, the upregulation of genes regulated by NF-kB in response to TNF was observed in all five cell types. Significant changes in key genes involved in AP-1 signaling were also observed. RBC contamination was a major issue in stored blood, whereas RBC adherence had no direct impact on the cell transcriptome. Conclusions: Significant transcriptome changes were observed across different PBMC cell types as a factor of time from blood draw to PBMC isolation and as a consequence of blood storage. This should be kept in mind when interpreting experimental results.

## 1. Introduction

Peripheral blood mononuclear cells (PBMCs) are readily acquired from patients’ blood samples, and have been widely used in the study of different human diseases [1], e.g., for addressing immunological issues in autoimmune or infectious diseases. However, there is often a time delay from blood collection to PBMC isolation during the sampling process, which is particularly a concern in large-scale multi-institutional consortium studies. The time delay prior to PBMC isolation may lead to significant changes in the transcriptome profiles of PBMCs, and confound the research results [1]. Single cell RNA-seq (scRNAseq) is an experimentally sensitive albeit powerful research tool enabling us to clarify the transcriptomes of specific cell types in different physiological situations and pathological processes [2]. The potential experimental bias related to the time delay prior to PBMC isolation may necessitate particular caution for the scRNAseq approach.

This study aims to examine the impact of the time delay prior to PBMC isolation on the transcriptome profiles of PBMCs in a scRNAseq study. Five major cell types in PBMCs with critical roles in innate, cellular, and humoral immunity were examined in this study, including CD4+ T cells, CD8+ T cells, natural killer (NK) cells, monocytes, and B cells. CD4+ T cells are the major components of PBMCs (25–60%) [3] and the major regulators of adaptive immune responses [4]. CD8+ T cells account for 5–25% of PBMCs [5], and develop into effector cells involving adaptive immune responses [6]. NK cells account for 10–15% of PBMCs [7], which are effector cells of the innate immune system, with critical roles in anti-viral infection and the regulation of autoimmunity [8]. Monocytes account for 10–20% of PBMCs [9], which are effector cells of the innate immune system and differentiate into macrophages and inflammatory dendritic cells (DCs) during inflammation responses [10,11]. B cells are in the range of 5–10% of PBMCs [9], and are the major regulators of humoral immunity of the adaptive immune system by producing antibodies [12]. Given their broad regulatory role in innate immunity and autoimmune/inflammatory diseases, transcriptome changes at different times of PBMC isolation and storage were assessed for each of these cell types, respectively.

## 2. Materials and Methods

### 2.1. Samples

Blood samples of 2 healthy adults (1 male and 1 female) were collected in EDTA-coated tubes. To assess the impact of prolonged storage of whole blood prior to PBMC isolation, one aliquot was processed immediately to isolate PBMCs by Ficoll density gradient centrifugation at the biorepository laboratory at the Center for Applied Genomics (CAG), the Children’s Hospital of Philadelphia (CHOP), while another aliquot was stored for 72 h at 4 °C prior to PBMC isolation following the same PBMC protocol. All isolated PBMCs were resuspended in freezing media and stored in liquid nitrogen.

### 2.2. Single Cell RNA-seq (scRNAseq)

scRNAseq in this study was done using 10X Chromium Single Cell 3′ Gene Expression Solution (10X Genomics, v3 chemistry) [13]. At the time of the experiment, cell suspensions were thawed, and cell aliquots were taken immediately for scRNAseq. Single-cell isolation and library preparation were performed at CAG, CHOP. Sequencing was performed using the Illumina Hiseq2500 SBS v4. The Chromium scRNAseq output data were processed using the Cell Ranger 7.1.0 analytical pipeline (10X Genomics), with reads aligned to the GRCh38 reference genome (Table 1). Low-quality cells with unique molecular identifiers (UMI) < 500 were removed from further analysis. Each cell type was filtered by log2-transformed value > 1 with the attribute parameter of Feature Max. With 10X Chromium being a highly sensitive genomic technology and where mRNAs with low expression levels create greater levels of noise [14], this study focused on the genes with relatively high expression levels, i.e., average occurrence greater than 1 count per cell across the entire dataset.

### 2.3. Data Analysis

Cell subtypes and differential expression (DE) were analyzed with Cell Ranger 7.1.0 (10X Genomics) and the Loupe Browser 6.2.0 (10X Genomics). Libraries were normalized for sequencing depth across all libraries during aggregation for DE analysis. Benjamini–Hochberg-corrected *p*-values were used to adjust for multiple testing and control the false discovery rate (FDR). FDR-corrected *p*-values < 0.1 were considered statistically significant. The DE comparison was done by comparing prior to and post blood storage within each individual, and then we combined the data by taking the average of the normalized counts of each gene in both subjects for Gene Set Enrichment Analysis (GSEA). The GSEA analysis was performed by the GSEA v4.3.2 software (Broad Institute of MIT and Harvard, Cambridge, MA, USA) based on the Molecular Signatures Database (MSigDB) [15] hallmark gene set collection [16]. The GSEA was based on all genes with an average occurrence greater than 1 count per cell across the entire dataset in fresh blood samples and blood samples stored for 72 h from both individuals.

## 3. Results

**Figure 1:**
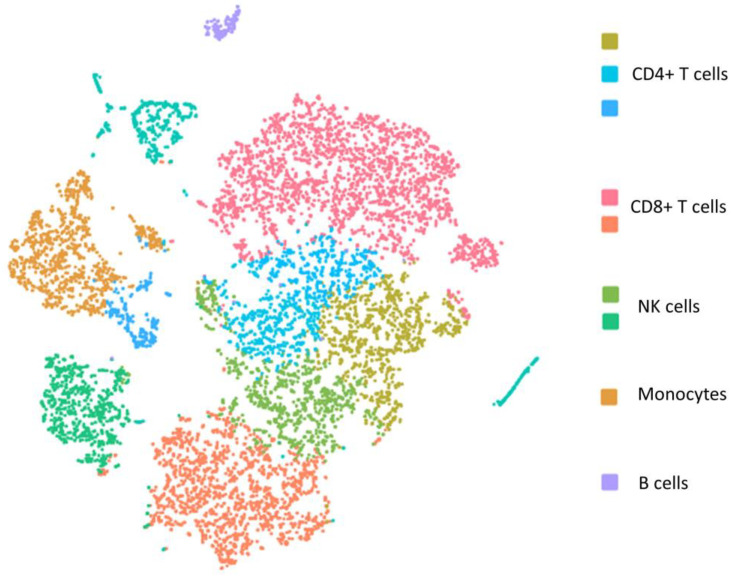
The t-distributed stochastic neighbor embedding (t-SNE) plot of single cell transcriptome of PBMCs isolated from fresh blood sample.

**Figure 2:**
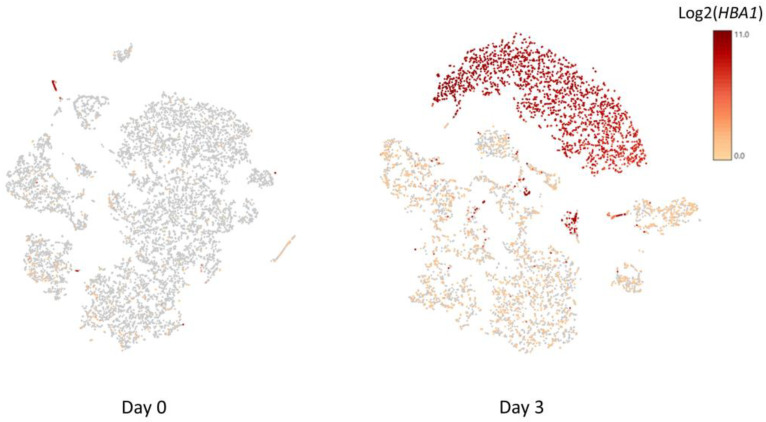
The t-distributed stochastic neighbor embedding (t-SNE) plot of single cell transcriptome of PBMCs isolated from fresh blood sample and stored blood sample. The color represents the expression of the hemoglobin subunit alpha 1 gene (*HBA1*).

Despite following the same PBMC isolation protocol for both fresh blood samples and samples stored for 72 h, yield rates of different cell types of PBMCs were significantly lower in the stored blood samples (Table 2). Compared to the fresh blood samples (Figure 1), contamination of red blood cells (RBC) in the isolated PBMCs was observed in the stored blood based on the hemoglobin subunit alpha 1 gene (*HBA1*) in both individuals (Figure 2). As shown, all five major subtypes of PBMCs, CD4+ T cells, CD8+ T cells, NK cells, monocytes, and B cells, demonstrated both common changes and cell-type-specific changes in the single cell transcriptome profiling.

### 3.1. GSEA Analysis

GSEA analysis highlighted that a number of MSigDB Hallmark gene sets [16] were significantly impacted in stored blood (FDR < 0.1, Table 3). In particular, the HALLMARK_TNFA_SIGNALING_VIA_NFKB gene set was upregulated in all five cell types; the HALLMARK_APOPTOSIS gene set was upregulated in CD4+ T cells and monocytes. In contrast, expression changes of the gene sets HALLMARK_MYC_TARGETS_V1 and HALLMARK_OXIDATIVE_PHOSPHORYLATION showed heterogeneity across different cell types. HALLMARK_MYC_TARGETS_V1 and HALLMARK_OXIDATIVE_PHOSPHORYLATION were downregulated with statistical significance in CD4+ T cells, while HALLMARK_MYC_TARGETS_V1 had positive ES scores in CD8+ T cells and B cells, and HALLMARK_OXIDATIVE_PHOSPHORYLATION had a positive ES in monocytes.

### 3.2. Specific DE Genes by Blood Storage

Individual DE genes with at least 1.5 fold change were examined. Table 4 shows the numbers of DE genes in each cell type. Among 24,800 genes in the scRNAseq assay, the DE genes in stored blood are significantly replicable in the two individuals, for both upregulated genes and downregulated genes in each cell type (with high statistical significance by Chi-square test, Table 4). The significant replicability demonstrated the validity of the observed effects of blood storage on mRNA levels. The expression levels and statistics of individual genes in each cell type are shown in the Supplementary Data S1–S5. As a powerful tool for transcriptome profiling at a single cell level, the scRNAseq assay demonstrated statistical power to identify DE genes by comparing multiple cells from each cell type in each sample. The statistical significances of the DE genes were adjusted for multiple comparisons. Genes with both fold change ≥1.5 and significantly corrected *p*-values are shown in Table 5.

### 3.3. CD4+ T Cells and RBC Adherence

With the contamination of RBCs as an issue in stored blood (Figure 2), it is important to investigate whether the above observed changes in transcriptome in PBMCs were related to the effects of RBC adherence on PBMCs. For this purpose, we investigated the effects of RBC adherence in CD4+ T cells by comparing the transcriptomes of CD4+ T cells with (log2 value of CD4 > 1 and log2 value of HBA1 > 1) vs. without (log2 value of CD4 > 1 and log2 value of HBA1 ≤ 1) the *HBA1* feature (Supplementary Data S6). GSEA analysis showed no significant Hallmark gene sets. Interestingly, the two upregulated gene sets, HALLMARK_TNFA_SIGNALING_VIA_NFKB and HALLMARK_APOPTOSIS, in PBMCs from stored blood have negative ES scores (i.e., no up-regulation) in CD4+ T cells with positive *HBA1* (Table 6), implying that the upregulation of these gene sets in stored blood was not due to RBC contamination. Except for the three hemoglobin genes, including *HBA1*, hemoglobin subunit alpha 2 (*HBA2*), and hemoglobin subunit beta (*HBB*), no other gene showed statistical significance when comparing the transcriptomes of CD4+ T cells with vs. without *HBA1* features.

## 4. Discussion

Both cell degeneration [17] and RBC contamination [18] in stored blood may explain the decreased yield rates of different cell types in both the female and the male samples. In addition, both common changes and cell-type-specific changes in the single cell transcriptome profiling over time were observed consistently in both samples.

### 4.1. Gene Sets in Different Cell Types

According to previous studies, both granulocyte activation [19,20] and RBC contamination [18] due to blood storage might cause upregulation of the HALLMARK_TNFA_SIGNALING_VIA_NFKB gene set, i.e., genes regulated by nuclear factor kappa B (NF-κB) in response to tumor necrosis factor (TNF). Granulocytes in stored blood are activated [20]. Granulocyte activation is correlated with activated TNFα signaling in different cell types [21], while all five cell types in our study included only monocytes that had upregulated HALLMARK_TNFA_SIGNALING_VIA_NFKB. Although a previous study suggested that RBC contamination might increase TNF expression by PBMCs [18], our study showed no direct effects of RBC adherence on the transcriptomes of CD4+ T cells.

The HALLMARK_MYC_TARGETS_V1 gene set includes a group of genes regulated by MYC [16], involved in cell cycle progression and cell proliferation [22]. The downregulation of this gene set shows statistical significance only in CD4+ T cells, but it has positive ES scores in the two types of effectors of the adaptive immune system, CD8+ T cells and B cells. CD4+ and CD8+ T Cells are differently programmed for proliferative responses [23]. Instead, proliferation of CD8+ T Cells and B cells relies on antigenic stimulation [23,24]. RBC contamination may suppress the proliferation of CD4+ cells [25], which is consistent with our observation of downregulated MYC target genes, while the inhibitive effect does not require direct adherence, as shown by the lack of difference in the comparison of the transcriptomes of CD4+ T cells with vs. without *HBA1* features. In contrast, as shown by our scRNAseq results, this gene set is not downregulated in CD8+ T cells and B cells in stored blood.

HALLMARK_OXIDATIVE_PHOSPHORYLATION includes a group of genes encoding proteins involved in oxidative phosphorylation and the citric acid cycle [16]. The downregulation of this gene set shows statistical significance only in CD4+ T cells, suggesting downregulated energy metabolism, which may be related to decreased glucose in stored blood [26]. Downregulation of energy metabolism may also be related to downregulated cell proliferation, implied by the downregulated HALLMARK_MYC_TARGETS_V1. However, the gene set HALLMARK_OXIDATIVE_PHOSPHORYLATION has a positive ES in monocytes, suggesting maintained energy metabolism.

### 4.2. Significant DE Genes

Hemoglobin genes, including *HBA1*, *HBA2*, and *HBB*, are commonly detected and shown to have upregulated expression in conjunction with different cell types, i.e., CD4+ T cells, CD8+ T cells, natural killer (NK) cells, and monocytes, which can be explained by the adherence of RBCs with these white blood cells (WBCs) in stored blood [27], though RBC ambient RNA may also contribute to these signals. However, upregulated *HBA1*, *HBA2*, and *HBB* were less significant with B cells, suggesting less RBC adherence. This inference is also consistent with the higher yield rates of B cells in stored blood than other cell types, as shown in Table 2.

Upregulated expression of Jun proto-oncogene AP-1 transcription factor subunit (*JUN*) is also commonly seen in different cell types. NF-κB controls the activation of activating protein 1 (AP-1) [28,29]. *JUN* encodes the AP-1 transcription factor c-JUN, which activates gene transcription in response to stimulation [30]. In addition to being a gene in the HALLMARK_TNFA_SIGNALING_VIA_NFKB gene set, *JUN* is also in the HALLMARK_APOPTOSIS gene set, with its demonstrated roles in the induction of apoptosis of T cells [31] and monocytes [32].

In addition to *JUN*, a number of genes showed significant DEs in CD4+ cells, e.g., the FosB proto-oncogene, AP-1 transcription factor subunit (*FOSB*), activating transcription factor 3 (ATF3), interleukin 1 beta (IL-1β, encoded by *IL1B*), and tribbles pseudokinase 1 (*TRIB1*). FosB dimerizes with c-JUN and forms the AP-1 complex [33]. ATF3 upregulated the expression of genes related to inflammation (e.g., the expression of TNFα) and apoptosis [34] by binding to the cyclic AMP response element (CRE) in promoters [35], or interacting with other transcription factors, e.g., AP-1 [36]. IL-1β may activate the AP-1 pathway [37], whereas the AP-1 signaling pathway also promotes *IL1B* transcription [38]. As a scaffold protein, tribbles pseudokinase 1 (*TRIB1*) increases NF-κB and AP-1 promoter activity through protein–protein interactions [39].

In B cells, significantly upregulated genes in the HALLMARK_TNFA_SIGNALING_VIA_NFKB gene set included early growth response 1 (*EGR1*), immediate early response 2 (*IER2*), dual specificity phosphatase 2 (*DUSP2*), and the CD83 molecule (*CD83*), in addition to *JUN*. The *EGR1* promoter is a target of *JUN* [40], while EGR1 has critical roles in both cell proliferation [41] and apoptosis [42] by transcriptional regulation. IER2 is also a component of the AP-1 transcription factor [43], and is involved in cell proliferation and apoptosis as an adaptor protein [44]. Transcription of *DUSP2* is regulated by AP-1, dephosphorylates mitogen-activated protein (MAP) kinases, and regulates cell proliferation and differentiation [45]. CD83 transcription is regulated by NF-κB [46], with higher expression on activated B cells [47], regulating the maturation and activation of B cells [48]. In contrast to the upregulated genes, two genes were significantly downregulated in B cells in stored blood, i.e., lysozyme (*LYZ*), encoding an antimicrobial agent [49], and S100 calcium binding protein A9 (*S100A9*), encoding a small calcium-binding protein as a potent stimulator of neutrophils [50]. Downregulation of these two genes suggested the inhibited effector function of B cells, in spite of the upregulated HALLMARK_TNFA_SIGNALING_VIA_NFKB genes.

In conclusion, compared to immediate isolation, we observed significant changes in the transcriptome profiles of multiple different cell types within the PBMC cell population upon 72 h blood storage prior to PBMC isolation. In particular, two well-pursued gene sets in PBMC studies, HALLMARK_TNFA_SIGNALING_VIA_NFKB and HALLMARK_APOPTOSIS, were upregulated in PBMCs extracted from blood stored for 72 h. Significant changes in key genes involved in AP-1 signaling were highlighted in CD4+ T cells and B cells. Considering the important roles of NF-κB and AP-1 signaling in the proliferation, function, and apoptosis of immune cells, as highlighted in numerous studies on human diseases in the literature, changes in the expression of these genes in stored blood warrants caution regarding experimental bias related to granulocyte activation and RBC contamination. With the scRNAseq technology as a highly sensitive and powerful tool, PBMCs extracted from fresh blood are needed for performing transcriptome studies, especially studies with a case–control design. With RBC contamination as a major issue in stored blood, as shown in this study, RBC adherence has no direct impact on cell transcriptome, as shown by our comparison of the transcriptomes of CD4+ T cells with vs. without the *HBA1* feature.

## Figures and Tables

**Table 1:** Cell number and read depth of each sample.

	Fresh Blood	Stored Blood
Female		
Estimated Number of Cells	8988	6047
Mean Reads per Cell	25,442	36,632
Male		
Estimated Number of Cells	9641	7088
Mean Reads per Cell	23,261	35,654

**Table 2:** Single cell transcriptome analysis in five types of white blood cells.

	Feature *	Fresh Blood (n)	Stored Blood (n)	Stored/Fresh Blood (%)
Female				
CD4+ T cells	*CD4 > 1*	763	248	32.5%
CD8+ T cells	*CD8A > 1*	912	440	48.2%
NK cells	*KLRB1 > 1*	2382	629	26.4%
Monocytes	*CD14 > 1*	756	212	28.0%
B cells	*CD79A > 1*	161	147	91.3%
Male				
CD4+ T cells	*CD4 > 1*	1344	195	14.5%
CD8+ T cells	*CD8A > 1*	457	73	16.0%
NK cells	*KLRB1 > 1*	1876	230	12.3%
Monocytes	*CD14 > 1*	1835	270	14.7%
B cells	*CD79A > 1*	419	100	23.9%

n: number of cells for single cell transcriptome analysis. * The cell-type-specific mRNA feature used to filter cells. Each cell type may be subjected to the nonspecificity of the cell marker.

**Table 3:** GSEA analysis of single cell transcriptome of five types of white blood cells.

Cell Type	HALLMARK Gene Set	SIZE	ES	NES	NOM *p*-Val	FDR q-Val
CD4+ T cells	HALLMARK_TNFA_SIGNALING_VIA_NFKB	63	0.81	2.25	<1 × 10^−4^	<1 × 10^−4^ *
	HALLMARK_APOPTOSIS	39	0.74	1.91	<1 × 10^−4^	6.69× 10^−4^ *
	HALLMARK_UV_RESPONSE_UP	28	0.78	1.91	<1 × 10^−4^	1.00 × 10^−3^ *
	HALLMARK_INFLAMMATORY_RESPONSE	38	0.68	1.81	1.36 × 10^−3^	0.005 *
	HALLMARK_KRAS_SIGNALING_UP	29	0.68	1.72	2.82 × 10^−3^	0.017 *
	HALLMARK_APICAL_JUNCTION	22	−0.58	−1.75	6.33 × 10^−3^	0.018 *
	HALLMARK_IL2_STAT5_SIGNALING	27	0.69	1.68	7.15 × 10^−3^	0.019 *
	HALLMARK_HYPOXIA	37	0.64	1.68	1.07 × 10^−2^	0.021 *
	HALLMARK_P53_PATHWAY	31	0.67	1.69	5.58 × 10^−3^	0.023 *
	HALLMARK_MYC_TARGETS_V1	66	−0.49	−1.78	6.29 × 10^−3^	0.026 *
	HALLMARK_OXIDATIVE_PHOSPHORYLATION	54	−0.44	−1.60	0.019	0.043 *
	HALLMARK_E2F_TARGETS	15	−0.62	−1.63	0.010	0.046 *
	HALLMARK_EPITHELIAL_MESENCHYMAL_TRANSITION	23	0.63	1.51	0.045	0.087 *
CD8+ T cells	HALLMARK_TNFA_SIGNALING_VIA_NFKB	28	0.77	2.01	<1 × 10^−4^	<1 × 10^−4^ *
	HALLMARK_P53_PATHWAY	16	0.64	1.51	0.053	0.140
	HALLMARK_ALLOGRAFT_REJECTION	32	−0.45	−1.32	0.107	0.394
	HALLMARK_HYPOXIA	20	0.50	1.20	0.268	0.421
	HALLMARK_OXIDATIVE_PHOSPHORYLATION	29	−0.40	−1.15	0.267	0.423
Monocytes	HALLMARK_TNFA_SIGNALING_VIA_NFKB	38	0.77	1.83	1.37 × 10^−3^	0.019 *
	HALLMARK_APOPTOSIS	18	0.84	1.84	4.73 × 10^−3^	0.026 *
	HALLMARK_APICAL_JUNCTION	16	−0.60	−1.56	0.027	0.070 *
	HALLMARK_MTORC1_SIGNALING	22	−0.49	−1.40	0.085	0.118
	HALLMARK_UV_RESPONSE_UP	17	0.75	1.64	0.060	0.118
NK cells	HALLMARK_TNFA_SIGNALING_VIA_NFKB	29	0.73	1.95	6.63 × 10^−3^	0.015 *
	HALLMARK_OXIDATIVE_PHOSPHORYLATION	26	−0.33	−1.14	0.273	0.553
	HALLMARK_P53_PATHWAY	18	0.49	1.21	0.276	0.586
	HALLMARK_INTERFERON_GAMMA_RESPONSE	19	0.38	0.96	0.431	0.670
	HALLMARK_ALLOGRAFT_REJECTION	34	0.30	0.82	0.567	0.690
B cells	HALLMARK_TNFA_SIGNALING_VIA_NFKB	27	0.85	2.04	<1 × 10^−4^	<1 × 10^−4^ *
	HALLMARK_ALLOGRAFT_REJECTION	21	−0.44	−1.02	0.440	0.434
	HALLMARK_P53_PATHWAY	16	0.54	1.19	0.308	0.464
	HALLMARK_INTERFERON_GAMMA_RESPONSE	17	0.50	1.09	0.392	0.477
	HALLMARK_HYPOXIA	17	0.54	1.20	0.263	0.669

* FDR < 0.1. ES, enrichment score; NES, normalized enrichment score; NOM, nominal; FDR, false discovery rate.

**Table 4:** Numbers of genes with at least 1.5 fold change by blood storage.

Count	Both Individuals	Female	Male	*p*-Value
All DE genes				
CD4+ T cells	72	111	215	<1 × 10^−303^
CD8+ T cells	25	61	102	1.9 × 10^−303^
Monocytes	29	50	67	<1 × 10^−303^
NK cells	6	39	33	1.3 × 10^−110^
B cells	51	159	62	<1 × 10^−303^
Up-regulated genes				
CD4+ T cells	64	97	93	<1 × 10^−303^
CD8+ T cells	18	50	23	<1 × 10^−303^
Monocytes	27	42	53	<1 × 10^−303^
NK cells	5	37	17	2.1 × 10^−146^
B cells	36	106	39	<1 × 10^−303^
Down-regulated genes				
CD4+ T cells	8	14	122	4.4 × 10^−120^
CD8+ T cells	7	11	79	1.6 × 10^−170^
Monocytes	2	8	14	2.76 × 10^−77^
NK cells	1	2	16	4.48 × 10^−28^
B cells	15	53	23	<1 × 10^−303^

**Table 5:** Genes with at least 1.5 fold change and significant *p*-values by blood storage.

Cell Type	Feature	Day 3 Average	Day 0 Average	Log2 Fold Change	*p*-Value	Day 3 Average	Day 0 Average	Log2 Fold Change	*p*-Value
		Female				Male			
CD4+ T cells	*AC007952.4*	2.30	0.99	1.21	0.060	2.73	0.61	2.15	1.98 × 10^−5^
	*ATF3*	2.04	0.45	2.17	4.74 × 10^−7^	2.32	0.78	1.57	7.30 × 10^−3^
	*CCL3*	2.01	0.27	2.88	4.93 × 10^−8^	3.85	0.54	2.83	5.85 × 10^−8^
	*CSRNP1*	1.57	0.54	1.55	1.12 × 10^−3^	1.50	0.52	1.51	0.015
	*CXCL8*	5.23	1.38	1.92	3.07 × 10^−4^	5.87	1.29	2.18	2.69 × 10^−4^
	*EREG*	2.11	0.39	2.42	5.71 × 10^−5^	1.60	0.32	2.30	6.30 × 10^−4^
	*FOSB*	13.49	4.67	1.53	8.43 × 10^−4^	9.85	4.07	1.27	0.091
	*HBA1*	5.88	0.04	7.19	5.66 × 10^−4^	17.34	0.90	4.26	2.02 × 10^−3^
	*HBA2*	12.17	0.05	8.00	5.38 × 10^−6^	41.63	2.18	4.25	6.30 × 10^−4^
	*HBB*	44.08	0.10	8.77	1.52 × 10^−4^	186.69	11.43	4.02	5.15 × 10^−3^
	*HES4*	1.46	0.33	2.12	1.98 × 10^−4^	1.22	0.44	1.46	0.091
	*IL1B*	8.73	2.19	1.99	1.22 × 10^−5^	10.23	2.18	2.22	4.49 × 10^−6^
	*JUN*	15.28	3.91	1.96	2.56 × 10^−6^	15.91	3.61	2.13	9.31 × 10^−6^
	*LUCAT1*	1.08	0.39	1.48	0.013	1.24	0.33	1.93	1.06 × 10^−3^
	*NFKBIZ*	3.75	1.69	1.14	0.062	4.08	1.71	1.25	0.091
	*OTUD1*	2.51	0.75	1.73	1.82 × 10^−4^	1.96	0.56	1.79	9.64 × 10^−4^
	*PPIF*	1.73	0.52	1.73	2.86 × 10^−4^	2.22	0.77	1.53	0.013
	*TRIB1*	1.72	0.73	1.23	0.043	1.76	0.70	1.33	0.069
Monocytes	*AC007952.4*	1.33	0.54	1.31	1.12 × 10^−3^	3.03	0.91	1.73	8.12 × 10^−6^
	*CCL3*	1.38	0.32	2.11	3.89 × 10^−9^	3.94	0.68	2.53	9.33 × 10^−12^
	*CXCL8*	2.53	0.79	1.68	3.88 × 10^−5^	7.52	2.32	1.69	4.63 × 10^−5^
	*HBA1*	12.55	0.40	4.99	4.20 × 10^−16^	20.33	0.18	6.80	1.54 × 10^−14^
	*HBA2*	25.33	0.68	5.22	1.12 × 10^−17^	50.72	0.48	6.72	2.78 × 10^−17^
	*HBB*	100.85	2.94	5.10	1.79 × 10^−15^	217.27	2.51	6.43	1.54 × 10^−14^
	*IL1B*	3.38	0.99	1.77	5.35 × 10^−7^	9.49	2.92	1.70	1.03 × 10^−5^
	*JUN*	12.17	3.41	1.83	1.06 × 10^−8^	15.89	3.63	2.13	4.69 × 10^−10^
	*OTUD1*	1.04	0.41	1.34	7.63 × 10^−4^	1.72	0.67	1.36	2.31 × 10^−3^
NK cells	*HBA1*	11.56	0.28	5.36	1.09 × 10^−20^	19.33	0.71	4.77	6.31 × 10^−6^
	*HBA2*	23.70	0.48	5.63	2.92 × 10^−23^	49.89	1.86	4.74	7.11 × 10^−7^
	*HBB*	97.34	2.11	5.53	4.97 × 10^−20^	221.54	9.03	4.61	1.72 × 10^−6^
	*JUN*	14.38	4.42	1.70	1.46 × 10^−7^	12.43	4.59	1.43	0.054
B cells	*AC103591.3*	2.31	0.58	1.99	3.33 × 10^−4^	1.16	0.26	2.14	7.27 × 10^−3^
	*AC253572.2*	1.75	0.13	3.76	7.10 × 10^−9^	1.79	0.29	2.63	3.02 × 10^−5^
	*CD83*	3.82	0.41	3.20	2.23 × 10^−13^	2.09	0.87	1.26	0.084
	*DUSP2*	2.95	0.28	3.37	1.16 × 10^−12^	2.01	0.36	2.46	1.58 × 10^−4^
	*EGR1*	2.33	0.17	3.78	3.62 × 10^−12^	1.08	0.21	2.37	2.73 × 10^−4^
	*IER2*	6.66	1.64	2.02	1.20 × 10^−6^	6.22	1.88	1.72	1.54 × 10^−3^
	*JUN*	17.86	4.21	2.08	1.84 × 10^−8^	13.28	5.48	1.27	0.055
	*LYZ*	0.01	3.24	−7.38	1.05 × 10^−4^	0.02	3.68	−6.96	2.73 × 10^−4^
	*S100A9*	0.01	5.73	−8.20	2.03 × 10^−3^	0.01	3.64	−7.36	0.018

**Table 6:** GSEA analysis of single cell transcriptome in CD4+ T cells with vs. without *HBA1* feature.

NAME	SIZE	ES	NES	NOM *p*-Val	FDR q-Val
HALLMARK_HEME_METABOLISM	22	0.73	1.90	0.092	0.298
HALLMARK_KRAS_SIGNALING_UP	32	0.34	0.87	0.435	1.000
HALLMARK_COAGULATION	15	0.31	0.82	0.520	0.846
HALLMARK_MTORC1_SIGNALING	46	0.21	0.54	0.845	1.000
HALLMARK_COMPLEMENT	42	0.21	0.53	0.826	0.882
HALLMARK_MYC_TARGETS_V1	70	−0.34	−1.22	0.172	1.000
HALLMARK_APOPTOSIS	42	−0.35	−1.17	0.234	1.000
HALLMARK_INFLAMMATORY_RESPONSE	44	−0.35	−1.16	0.244	1.000
HALLMARK_APICAL_JUNCTION	22	−0.39	−1.14	0.296	1.000
HALLMARK_INTERFERON_ALPHA_RESPONSE	21	−0.38	−1.09	0.371	1.000
HALLMARK_OXIDATIVE_PHOSPHORYLATION	57	−0.31	−1.09	0.335	1.000
HALLMARK_IL2_STAT5_SIGNALING	30	−0.34	−1.04	0.428	1.000
HALLMARK_EPITHELIAL_MESENCHYMAL_TRANSITION	24	−0.34	−1.01	0.458	1.000
HALLMARK_GLYCOLYSIS	19	−0.34	−0.95	0.547	1.000
HALLMARK_UV_RESPONSE_UP	29	−0.30	−0.91	0.600	1.000
HALLMARK_ALLOGRAFT_REJECTION	50	−0.27	−0.89	0.648	1.000
HALLMARK_UNFOLDED_PROTEIN_RESPONSE	22	−0.30	−0.88	0.664	1.000
HALLMARK_E2F_TARGETS	17	−0.32	−0.87	0.685	1.000
HALLMARK_TNFA_SIGNALING_VIA_NFKB	72	−0.25	−0.87	0.712	1.000
HALLMARK_G2M_CHECKPOINT	25	−0.26	−0.76	0.825	1.000
HALLMARK_HYPOXIA	38	−0.23	−0.75	0.852	1.000
HALLMARK_INTERFERON_GAMMA_RESPONSE	52	−0.22	−0.73	0.898	1.000
HALLMARK_ADIPOGENESIS	29	−0.23	−0.72	0.855	1.000
HALLMARK_PI3K_AKT_MTOR_SIGNALING	23	−0.25	−0.72	0.840	1.000
HALLMARK_ANDROGEN_RESPONSE	17	−0.27	−0.71	0.841	1.000
HALLMARK_P53_PATHWAY	34	−0.23	−0.70	0.879	1.000
HALLMARK_IL6_JAK_STAT3_SIGNALING	15	−0.24	−0.64	0.904	1.000
HALLMARK_MITOTIC_SPINDLE	19	−0.21	−0.58	0.943	1.000
HALLMARK_ESTROGEN_RESPONSE_LATE	17	−0.19	−0.52	0.978	0.988

ES, enrichment score; NES, normalized enrichment score; NOM, nominal; FDR, false discovery rate.

## Data Availability

Supporting data from this study can be obtained by emailing the corresponding author, Dr. Hakon Hakonarson.

## Supplementary Materials

The following supporting information can be downloaded at: https://www.mdpi.com/article/10.3390/genes14010142/s1, Supplementary Data S1: List of genes in CD4+ T cells. Supplementary Data S2: List of genes in CD8+ T cells. Supplementary Data S3: List of genes in NK cells. Supplementary Data S4: List of genes in monocytes. Supplementary Data S5: List of genes in B cells. Supplementary Data S6: List of genes affected by HBA1 in CD4+ T cells at Day 3.
